# Discrete blue and green light wavebands alter biomass, morphology, and color in lettuce (*Lactuca sativa L.)*

**DOI:** 10.3389/fpls.2026.1735363

**Published:** 2026-05-05

**Authors:** Masaki Kurosaki, Christopher P. Levine, Neil S. Mattson

**Affiliations:** 1 School of Integrative Plant Science, College of Agriculture and Life Sciences, Cornell University, Ithaca, NY, United States; 2 Crop Physiology Laboratory, Department of Agricultural and Environmental Biology, Graduate School of Agricultural and Life Sciences, The University of Tokyo, Tokyo, Japan

**Keywords:** blue light, controlled environment agriculture, green light, lettuce, photomorphogenesis, wavebands

## Abstract

Previous research demonstrated that blue (B) light decreased internode elongation and leaf surface area (LSA) while increasing anthocyanin concentration as the red (R) to B ratio decreased, but the effects of specific B wavebands on lettuce remain underexplored. The first experiment objective was to evaluate the impacts of three different wavebands of B light (peaks and full width at half max [FWHM]: 412 nm, FWHM 16 nm; 425 nm, FWHM 15 nm; 454 nm, FWHM 20 nm) and green (G) light (523 nm, FWHM 34 nm) on ‘Rex’ and ‘Red Oak’ lettuce biomass and morphology. Among B light treatments, 412 and 425 nm produced similar results, with significantly greater growth (width, fresh weight (FW), leaf area (LA) for both cultivars, height and dry weight (DW) for ‘Rex’) compared to the 454 nm treatment. 454 nm treatment showed more intense characteristics associated with B light exposure (decreased height, width, LA, FW, and DW) but had the highest chlorophyll content. ‘Red Oak’ anthocyanin concentration was unaffected by B wavelengths. Plants under G light had similar biomass to 412 and 425 nm B light for ‘Rex’ and 412 nm for ‘Red Oak’ but lower chlorophyll and anthocyanin concentration. The second objective of this study was to assess whether G light’s biomass could be complemented by B light’s pigmentation effects when G was replaced by B late in the crop cycle. The control treatment used 80% R and 20% G (523 nm), with G substituted by 454 nm B light 2, 4, or 8 days before harvest. Two days of B light at the end of the cycle resulted in similar FW as the control in ‘Red Oak’ but significantly higher anthocyanin. In ‘Rex’, FW and DW were statistically unchanged, but longer B light exposure significantly increased chlorophyll content. This indicated that two days of B light at the cycle’s end preserved biomass and improved crop quality through enhanced leaf coloration in both cultivars.

## 1 Introduction

Light emitting diodes (LEDs) offer precise control over light spectrum and intensity, unlike high-intensity discharge (HID) lighting systems (Hernandez et al., 2020; Nelson and Bugbee, 2014). Unlike HIDs, LEDs emit minimal infrared radiation, reducing leaf surface warming (Dueck et al., 2012). This can be beneficial in warm conditions but less so in cooler environments (Lee et al., 2020). The lower heat output also allows LEDs to be placed closer to or within the canopy, enhancing photon capture (Gómez and Mitchell, 2014; Nelson and Bugbee, 2014).

Light quality modulation can also be used to impact development and productivity (Both, 1995; Zhang et al., 2019). For example, supplementing or substituting far-red (FR) with PAR photons (modifying the proportion of FR in the total photon flux density (TPFD) across the 400–800 nm range) can, in some particular instances, be used to trigger shade-avoidance responses (i.e., stem elongation, increased canopy surface area, and thinner leaves) (Kusuma and Bugbee, 2023; Levine et al., 2026) but this is also temperature dependent (Jeong et al., 2024). In general, B light is known to trigger many physiological reactions including inhibiting stem elongation and causing greater anthocyanin pigmentation (Ahmad et al., 1995). There are two main photoreceptors sensing B light that cause those reactions, Phototropins and Cryptochrome. Phototropins regulate phototropism (plant orients itself to face the light source), stomatal opening, and chloroplast relocation within cells. Cryptochrome regulates stem elongation, pigmentation and senses the photoperiod (Chia and Kubota, 2010; Taiz et al., 2018). The degree of response often depends on the proportion of B photons within the total light intensity, and differs by species (Dougher and Bugbee, 2001). For lettuce, responses to B light were reported by various researchers. For lettuce plants under a photosynthetic photon flux density (PPFD) of 70 µmol·m^-2^·s^-1^, the hypocotyl length and leaf area (LA) decreased while the concentration of anthocyanin (R pigment) increased as the proportion of B light increased (Lee et al., 2010). When 130 µmol·m^-2^·s^-1^ of 476 nm peak B LED light was supplemented with cool white (CW) fluorescent light (total PPFD of 300 µmol·m^-2^·s^-1^) similar plant responses were induced such as higher anthocyanin concentration and shorter stem length compared with CW lighting alone or CW lighting plus substituted R or G light supplementation for B light at the same PPFD (Li and Kubota, 2009).

G photons are usually thought of as not as efficiently used for photosynthesis. However, based on the quantum yield work of McCree (1972), there is only a 20% lower efficiency for photosynthesis vs. R or B peaks. G photons are also an important factor for human vision because (Takao et al., 2016) mentioned possible health effects of monochromatic light. Lack of G light also makes it harder for the human eye to assess plant health or spot pests and nutritional deficiences. In addition, while R and B photons are absorbed by chloroplasts on adaxial surface of a leaf, G photons penetrate the leaf deeper and are absorbed on the abaxial surface and lower leaves (Brodersen and Vogelmann, 2010). This property is important for plant growth. For example, under high white (W) light intensity which typically has more G photons than typical mono or dichromatic R and B photons, some chloroplasts relocated to the abaxial surface of the leaf, and in this region G light was more important for photosynthesis than R light (Terashima et al., 2009). An experiment comparing red:blue = 84:16 and red:blue:green = 61:15:24 (substitution of some R light to G light) with 150 µmol·m^-2^·s^-1^ PPFD found that the supplemental G light treatment resulted in significantly higher shoot fresh weight (FW) (Kim et al., 2004).

While the effects of B and G light on lettuce are known, few studies compare specific B wavelengths. Planck’s equation states that the energy of a photon equals Planck’s constant multiplied by the speed of light divided by its wavelength. Shorter-wavelength blue photons carry more energy than longer-wavelength blue photons. In other words, within the blue region, a photon at around 430 nm has greater energy per photon than one at around 440 nm because energy increases as wavelength decreases. Furthermore, Ashenafi et al. (2023); Alrajhi et al. (2023), and Mohamed et al. (2021) collectively demonstrate that distinct regions within the blue spectrum can differentially influence plant morphology, physiology, and photosynthetic regulation. One study Samuolienė et al. (2011) assessed antioxidant and nutritional traits under different B or G wavelengths but did not report biomass. Samuolienė et al. (2011) used 16 h of supplemental B (455 or 470 nm) or G light (505 or 590 nm) at 30 µmol·m^-2^·s^-1^ with a background of supplemental HPS light providing 170 µmol·m^-2^·s^-1^ of PPFD in the greenhouse. Recent work in *in vitro* systems has also demonstrated that optimization of B and R spectral compositions and intensity can significantly influence morphological and physiological traits highlighting the growing interest in fine-tuned LED waveband applications beyond broad waveband categories (Sarropoulou et al., 2024, 2025). Anthocyanin content of red leaf ‘Multired 4’ lettuce was statistically higher under both B lighting treatments than HPS only or G lighting treatments (Samuolienė et al., 2011). Li et al. (2025) found 660 nm narrow band laser diode (LD) R promoted effective quantum yield of photosystem II (Y(II)), fraction of open photosystem II reaction centers (qL), starch accumulation, and photosynthetic capacity in arabidopsis and tobacco leaves. Although Li et al. (2025) does not demonstrate with B wavebands, it nonetheless shows increased capabilities of narrow waveband lighting similar to different blue wavebands.

Overall, more information on the growth responses of lettuce to B light peak wavelengths and wavebands could help lighting manufacturers select specific LEDs for their fixtures and help growers select an appropriate light spectrum for their crops. Recent work has begun to explore strategic light adjustments at the end of production, with studies such as Min et al. (2021) and Zhu et al. (2024) demonstrating that end of production (EoP) spectral shifts to higher energy photons can modulate plant morphology and biochemical composition, supporting the potential for targeted EoP light treatments to refine crop outcomes. Blue light can enhance morphology and quality traits, but prolonged high blue exposure during production may reduce biomass and increase energy costs. Therefore, applying blue light specifically as an EoP treatment allows growers to gain its benefits late in the cycle while avoiding growth penalties and unnecessary energy use earlier. The first objective was to assess how different B wavelengths and only one G peak wavelength, combined with 80% 661 nm R light, affect lettuce growth under sole-source lighting. The second objective was to determine how the timing of substituting G with B light impacts lettuce yield and quality.

We hypothesized that discrete B peak wavelengths would elicit distinct growth responses due to differences in cryptochrome activation, with 454 nm expected to induce stronger photomorphogenic inhibition than 412 or 425 nm. We also considered that the higher photon energy of shorter wavelength blue light could contribute to different physiological responses.

## 2 Material and methods

In this experiment, two lettuce cultivars, ‘Rex’ and ‘Salanova Red Oakleaf’, (Johnny’s Selected Seeds, Winslow, ME, USA) were selected as examples of a green butterhead and a red salad bowl type lettuce, respectively, that are currently prevalent in North American commercial CEA operations according to personal communications with U.S. CEA growers. The experiment was conducted in a walk-in controlled environment growth chamber with internal dimensions of 132”x 86”x 96”. The chamber contained a central aisle and two opposing plastic cultivation benches positioned approximately at waist height. The four lighting treatments were spatially separated with a black cloth in the middle aisle and with insulation board between the lights. Four independent treatment areas were within the single chamber that avoided cross contamination of spectral environments. After the seedling stage, plants were grown under four different lighting treatments set up in a controlled environment chamber. For experiment 1, the effects of either a B-enriched or G-enriched spectrum were evaluated. In experiment 2, plants were grown under a G spectrum, and then G light was substituted with B light for 2, 4, or 8 days at the end of the crop cycle to assess EoP responses.

### 2.1 Temperature and relative humidity conditions

During both experiments 1 and 2, the controlled environment chamber was maintained so that in each of the four lighting treatment areas, air temperature and relative humidity were 22/20°C day/night, and 70 ± 10%, respectively. The growing environment was monitored using a mini digital min/max thermometer, which was checked daily. Temperature was maintained at the respective temperature setpoints via thermostat control. For dehumidification control, a dehumidifier (model: 53RJ38, Dayton Electric Co., Dayton, TX, USA) was used.

### 2.2 Plant cultivation

A combination of equal parts Jack’s Hydroponic 5N-4.8P-21.6K (J.R. Peter’s Inc., Allentown, PA, USA) and YaraLiva Calcium Nitrate (Yara International ASA, Oslo, Norway) was the nutrient solution used to fertilize plants according to Mattson and Peters (2014) resulting in a final nutrient concentration (mg·L^-1^) of 159 N, 41 P, 168 K, 147 Ca, 49 Mg, 66 S, 2.33 Fe, 0.39 Mn, 0.12 Zn, 0.39 B, 0.12 Cu, and 0.15 Mo. In the reservoir, the pH and electrical conductivity (EC) were monitored weekly and adjusted as needed to be maintained at 5.8-6.0 and 1.6-1.7 dS·m^-1^, respectively.

A rockwool sheet (Grodan, Roermond, Netherlands) consisting of 200 2.5 × 2.5 cm cells was soaked in the nutrient solution for 15 minutes to condition. The rockwool was allowed to drain to container capacity. Then 100 seeds of each cultivar were sown in the rockwool sheet placed in a standard 1020 plastic tray (TO Plastics, Clearwater, MN, USA) and covered with a clear plastic humidity dome (Curtis Wagner Plastics Corp, Houston, TX, USA). Plants were grown in a seedling mini chamber with a T5 fluorescent (CW spectrum) light for 18.5 hours per day at an average PPFD of 300 µmol·m^-2^·s^-1^. Air temperature was maintained at 22/20°C (D/N). Two days after sowing, the humidity dome was removed. During the seedling stage, the flat was sub-irrigated daily, or as needed, with the nutrient solution. Eleven days after sowing, seedlings were transplanted into a commercial peat-lite mix (LM-111, Lambert, Québec, Canada) in 10-cm (473 mL volume) pots. Prior to transplanting the substrate was well watered with the nutrient solution. A total of 44 plants, 24 ‘Red Oak’ and 20 ‘Rex’, in which the 4^th^ true leaf had just emerged, were selected based on uniformity and transplanted. Under each lighting treatment area consisted of 33 cm x 55 cm lighted growing areas, 11 plants total were placed per treatment per cycle, six ‘Red Oak’ and five ‘Rex’, in the area marked under each light fixture mounted in each of 4 growth chamber sections. To maintain consistent irrigation across treatments, all pots were top-watered with about 50 mL at each watering event, and all treatments received the same volume of water simultaneously, applied daily or as needed.

### 2.3 Experiment 1 discrete blue and green light effects lighting treatments

**Figure 1:**
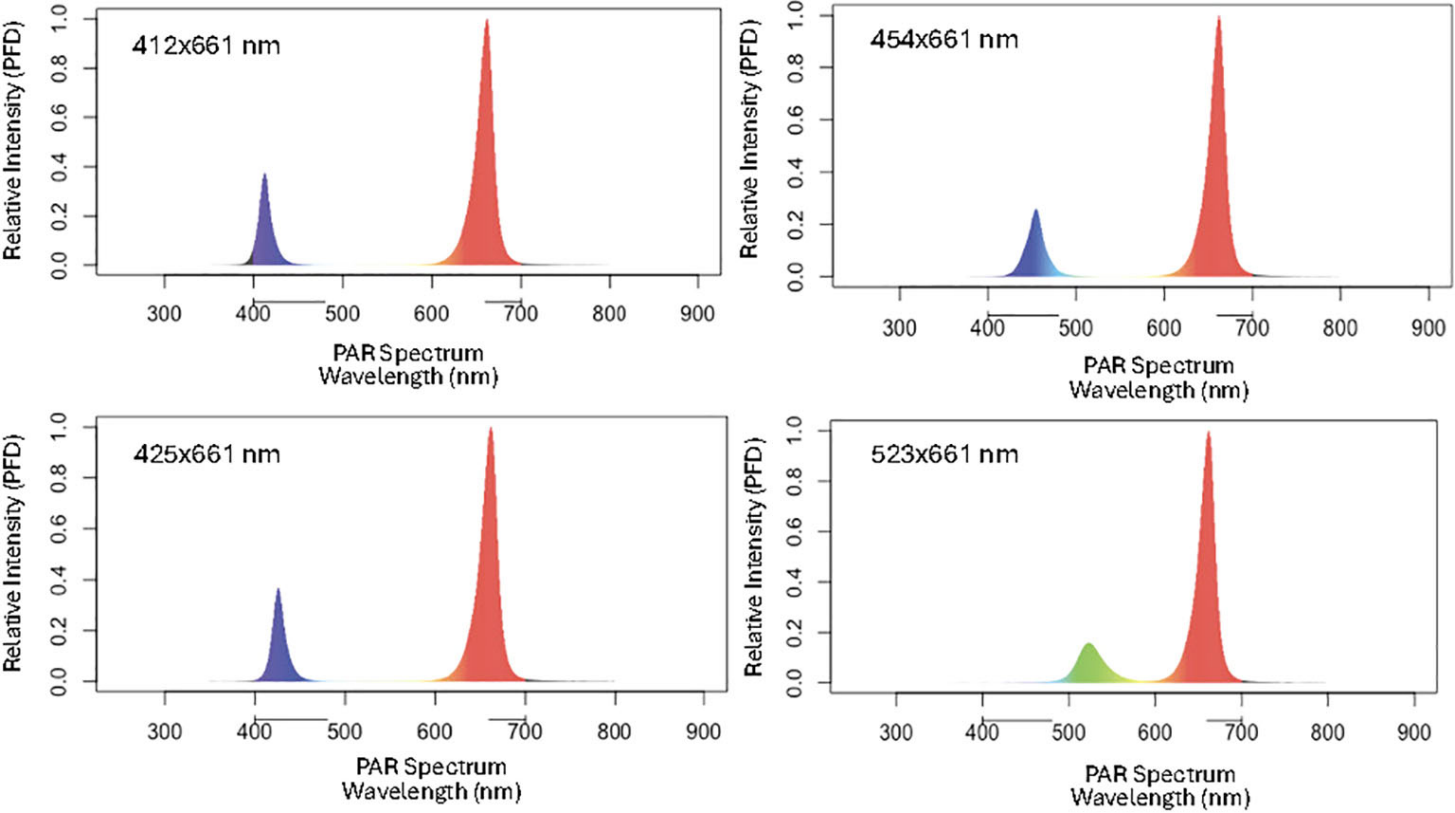
Light spectrum delivered to each treatment.

**Figure 2:**
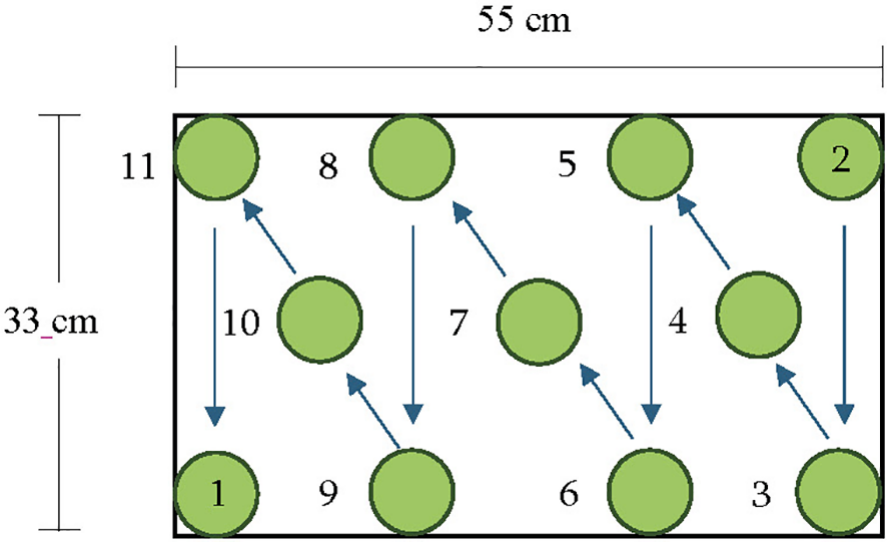
Location of plants and daily rotational movement across the lighting area. Arrows indicate the sequence of plant movement among positions. Representative image not to scale. Plant density was 60 plants m^2^.

There was one light fixture for each of the four treatment areas. Four 9 channel LED light fixtures: DYNA (Heliospectra AB, Gothenburg, Sweden) were used to deliver the lighting treatments. The 9 channels include: UV-A (380 nm), B (400 nm, 420 nm, 450 nm), G (530 nm), R (630 nm, 660 nm), FR (735 nm) and W (5700K). The listed wavelength peaks were provided by the company, however the spectral output of the relevant channels for this study was measured independently. The fixtures had an adjustable control system whereby each channel can be dimmed from 0 to 1000. The efficacy of each channel is shown in **Supplementary Table 1**. Experiment 1 was designed to test the effect of different wavebands of B or G light. During experiment 1, 80% of total PPFD was from 661 nm peak R (and 21 nm FWHM) and the remaining 20% came from treatments of 412 nm (16 nm FWHM), 425 nm (15 nm FWHM), 454 nm (20 nm FWHM) and 523 nm (34 nm FWHM) wavebands (**Supplementary Table 1**, **Figure 1**). Light quality was measured with a PS-300 spectroradiometer (Apogee Instruments, Inc., Logan, UT, USA). The lighting fixtures were hung 20 cm above the top of the spectroradiometer sensor (representing the top of the plant canopy) and a 1650 cm^2^ (30x55 cm) area was used for the treatment area. There were eleven plant positions and PPFD was measured for each spot for each of the four lighting treatments. The average mean PPFD under each lighting treatment was 255 µmol·m^-2^·s^-1^ (**Supplementary Table 2**). Each lighting system’s timer was set to light for 18.5 hours per day which resulted in a 17 mol·m^-2^·d^-1^ daily light integral (DLI). PPFD varied from 150 µmol·m^-2^·s^-1^ at perimeters of the lit area to 390 µmol·m^-2^·s^-1^ directly underneath the fixture. To account for variability in light intensity distribution under each fixture, plant position was rotated daily. Plant position was changed every night after lights were off following the arrows in **Figure 2**. (The plant in the position 1 was moved to position 2, etc. Since there were 11 plant positions, after 11 days, plants were back at their initial position and therefore experienced all positions a total of two times since the experiment was run for 22 days after transplanting for each crop cycle. This also resulted in a planting density of 60 plants ·m^2^.

### 2.4 Experiment 2 end-of-production blue light substitution lighting treatments

The objective of experiment 2 was to determine whether the growth benefits due to G light could be enhanced with darker pigmentation resulting from B light. The B light was provided 2, 4, or 8 days before the end of the crop cycle. The G light treatment was provided at the same PPFD, total average PPFD at 255 µmol·m^-2^·s^-1^, with R (661 nm, FWHM 21 nm) providing 80% of the PPFD and G (523 nm, FWHM 34 nm) providing 20% of the PPFD. This was provided as the base lighting treatment for all four treatments. Control plants received the 80% R, 20% G light throughout the 22-day crop cycle. For the other treatments, the portion of G light was substituted with B light (454 nm, FWHM 20 nm) for the last 2, 4 or 8 days of the crop cycle. B light was also provided at the same light intensity as the G, and a fixed R to B ratio (80% R and 20% B) was maintained. Each lighting system’s timer was set to light for 18.5 hours resulting in a 17 mol·m^-2^·d^-1^ DLI. PPFD varied from 150 µmol·m^-2^·s^-1^ at perimeters of the lit area to 390 µmol·m^-2^·s^-1^ directly under the fixture. To account for variability in light intensity distribution under each fixture, plant position was moved daily as described in **Figure 2**.

### 2.5 Plant measurements and statistical analysis

22 days after transplanting, plants were harvested and height (from soil line to top of the plant), width (average of two measurements above the plant: at the widest part of the plant, and at a 90° angle to the widest part), SPAD chlorophyll index (Chlorophyll Meter SPAD-502, Konica Minolta Inc., Tokyo, Japan) (an average of 3 measurements from 3 recently matured leaves on each plant) and L*a*b colorimeter (NR20XE, Shenzhen 3nh Technology CO., Shenzhen, China) (average of 3 measurements from 3 recently matured leaves on each plant) were collected. SPAD measurements were taken on the leaf blade while avoiding the major veins to ensure consistent and reproducible readings. The plants were then severed at the soil line to determine FW. Leaves were detached from the plant and leaf surface area was measured using LI-3100C Area Meter (LI-COR Inc., Lincoln, NE, USA). Three leaves were sampled from each ‘Red Oak’ plant and frozen immediately in liquid nitrogen and stored in -80°C freezer for the anthocyanin content analysis by UV-vis spectrophotometer (UV-1800, Shimadzu, Kyoto, Japan). Plants were placed in paper bags and placed in an oven (70°C for a week) for dry weight (DW) measurements. Anthocyanin content analysis utilized the methodology of (Neff 1998) and (Carvalho 2014) comparing relative anthocyanin levels. First, frozen leaves were ground, and 50 mg was placed in a microfuge tube. The sample was dissolved into methanol-HCl solution (99% MeOH, 1% HCl) and kept in a 4°C dark refrigerator overnight. Next, H_2_O (including a 40% by volume methanol-HCl solution) and chloroform were added into the tube resulting in separation of the aqueous (containing anthocyanin and other water soluble compounds) and precipitate (containing immiscible organic solvents, chlorophyll, carotenoids and lipids etc.) phases. After centrifuging, 400 µL of supernatant solution was removed and diluted by adding 2600 µL of water-methanol-HCl solution (40% H_2_O, 60% MeOH and 1% HCl). As reference the same volume (3000 µL) of water-methanol-HCl solution without sample was used. Absorbance of each tube at 530 and 657 nm was recorded, and anthocyanins were calculated following Equation 1 published in the literature (Carvalho and Folta, 2014; Neff and Chory, 1998).

### 2.6 Relative anthocyanin concentration

There were five replicate plants for both experiments one and two of ‘Rex’ and six replicate plants of ‘Red Oak’ under each lighting treatment. Four replicate crop cycles were conducted over time, with the lighting treatments rotated after each crop cycle so that each treatment was applied once in each of the four quadrants of the growth chamber. The relative anthocyanin concentration was calculated using the following equation: Relative anthocyanin concentration $=\left(Abs_{530}-Abs_{657}\right)*1000*{\left(powder\;weight\;of\;leaves\;\left(mg\right)\right)}^{-1}$. The collected data was analyzed in JMP version 13.1 (JMP Statistical Discovery LLC, Cary, NC, USA) using ANOVA to determine effect of crop cycle and lighting treatment. A Tukey Kramer honest significant difference (HSD) test at *α* = 0.05 was used to compare lighting treatments for each of the measured parameters.

## 3 Results

### 3.1 Discrete blue and green light effects on shoot height and width

**Figure 3:**
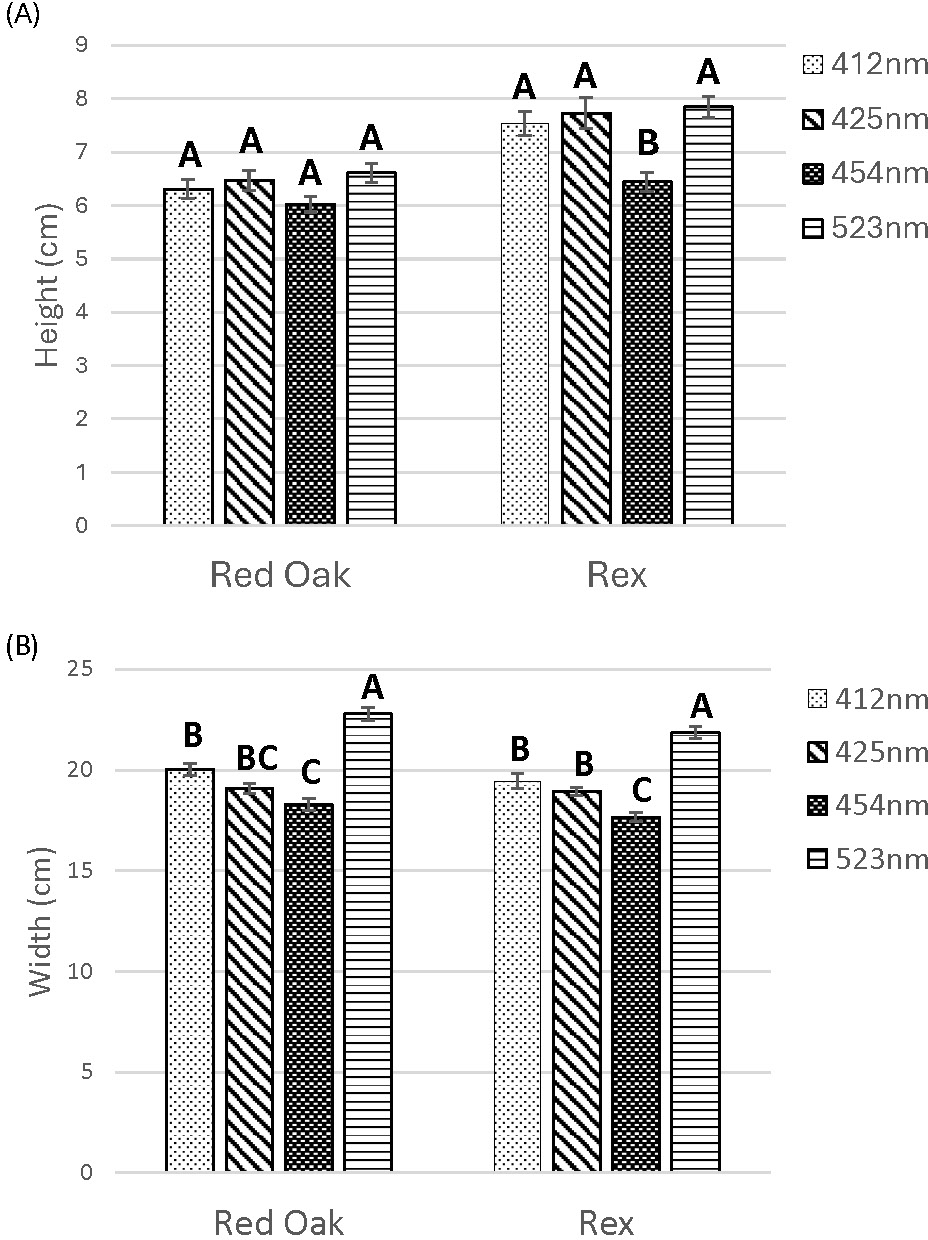
Shoot height **(A)** and width **(B)** of lettuce ‘Red Oak’ and ‘Rex’ after 22 days of sole-source lighting treatments. Plants received 80% of their light from red (661 nm, 21 nm FWHM) and 20% of their light from either 412 nm (16 nm FWHM), 425 nm (15 nm FWHM), 454 nm (20 nm FWHM) and 523 nm (34 nm FWHM) light. Data represent means (± SE) of 5 or 6 plants per cultivar per treatment times 4 crop cycles. Letters represent mean separation comparison across lighting treatments within a cultivar using Tukey’s HSD (α = 0.05).

**Figure 4:**
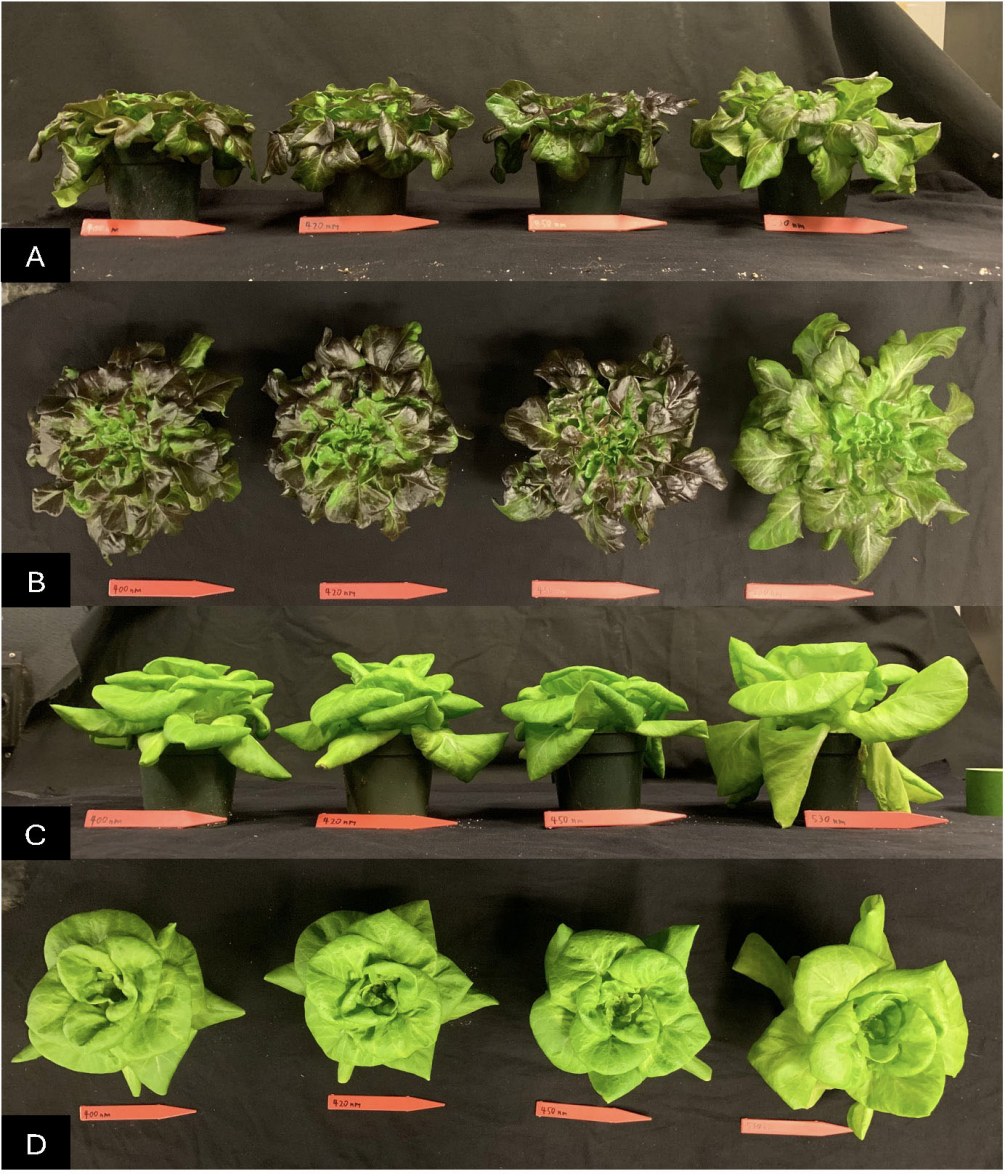
The appearance of representative ‘Red Oak’ from a side view **(A)**, from the top **(B)** and ‘Rex’ side view **(C)** and top view **(D)** after a 22 day crop cycle under light treatments which contained 20% (from left to right) peak wavelengths of 412, 425, 454 and 523 nm.

Lighting treatments did not impact shoot height of ‘Red Oak’ lettuce (**Figure 3A**). For ‘Rex’, the 454 nm treatment led to significantly shorter plant height than other wavelengths. (**Figures 3A**, **4A, C**).

For both cultivars of lettuce, the 523 nm lighting treatment resulted in the widest heads. The 412 nm treatment has smaller head width than 523 nm and there was also a pattern where increasing wavelength of B from 412 to 454 nm also led to decreasing plant width (**Figures 3B**, **4B, D**).

### 3.2 Discrete blue and green light effects on shoot FW and DW

**Figure 5:**
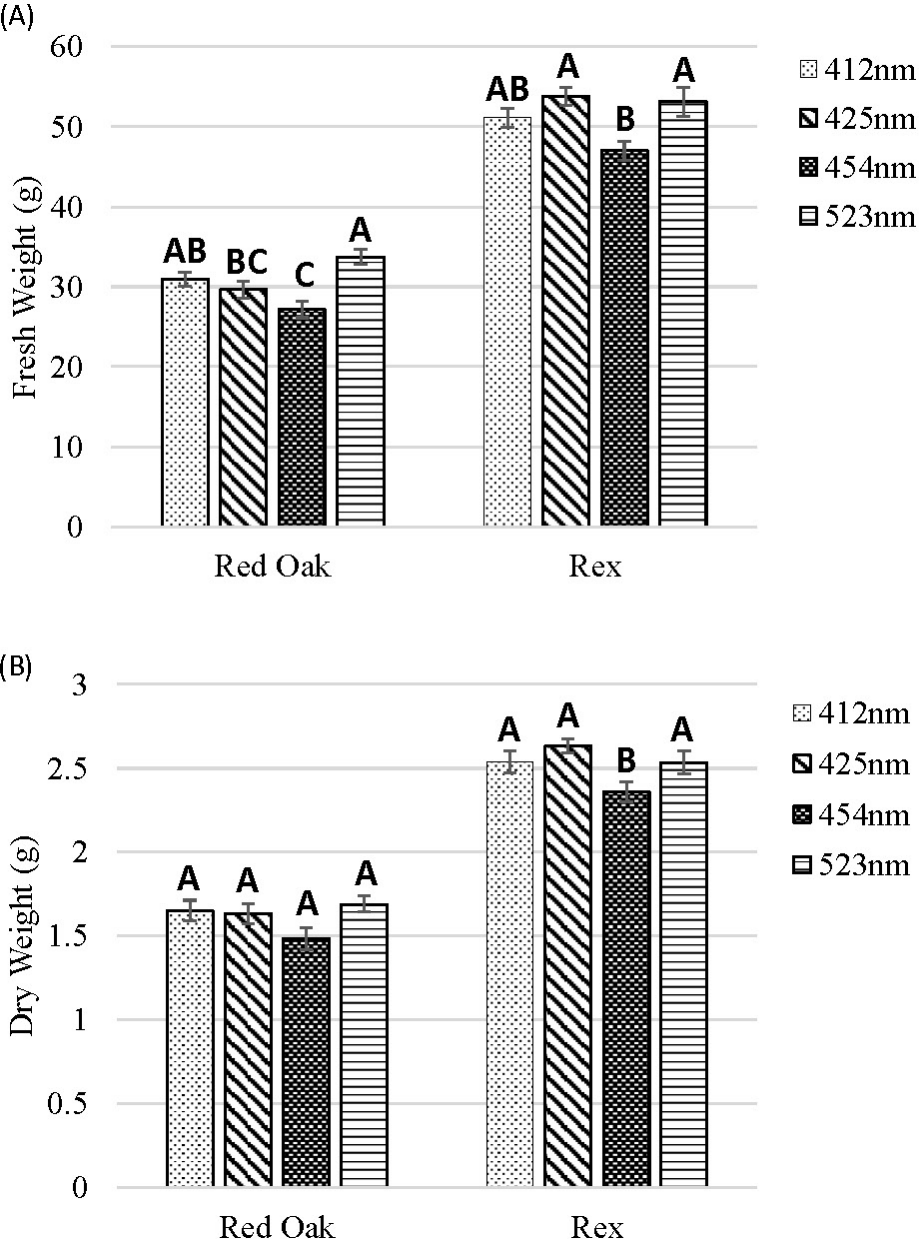
Shoot FW **(A)** and DW **(B)** of lettuce ‘Red Oak’ and ‘Rex’ after 22 days of sole-source lighting treatments. Plants received 80% of their light from R (661 nm, 21 nm FWHM) and 20% of their light from either 412 nm (16 nm FWHM), 425 nm (15 nm FWHM), 454 nm (20 nm FWHM) and 523 nm (34 nm FWHM) light. Data represent means (± SE) of 5 or 6 plants per cultivar per treatment times 4 crop cycles. Letters represent mean separation comparison across lighting treatments within a cultivar using Tukey’s HSD (α = 0.05).

For ‘Red Oak’, the greatest shoot FW (biomass) was found for the 523 and 412 nm treatments and increasing the wavelength of B light led to reductions in FW. For ‘Rex’, plants under the 454 nm treatment had smaller FW than under the 425 and 523 nm treatments (**Figure 5A**).

There were no significant differences in shoot DW biomass among treatments for ‘Red Oak’. For ‘Rex’, plants under the 454 nm treatment had the smallest DW (**Figure 5B**).

### 3.2 Discrete blue and green light effects on LA and specific leaf area

**Figure 6:**
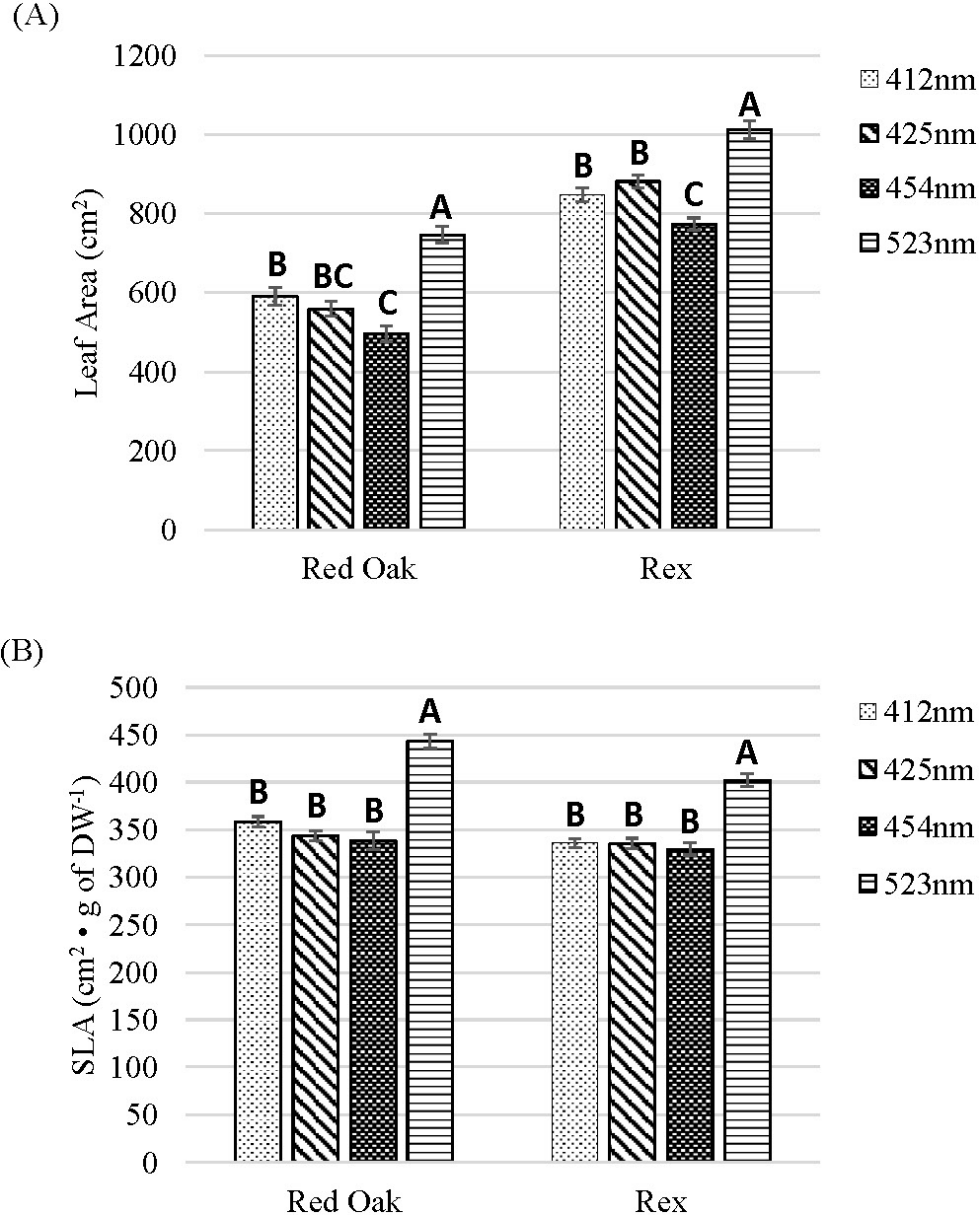
Leaf area (LA; **(A)**) and specific leaf area (SLA; **(B)**) of lettuce ‘Red Oak’ and ‘Rex’ after 22 days of sole-source lighting treatments. Plants received 80% of their light from R (661 nm, 21 nm FWHM) and 20% of their light from either 412 nm (16 nm FWHM), 425 nm (15 nm FWHM), 454 nm (20 nm FWHM) and 523 nm (34 nm FWHM) light. Data represent means (± SE) of 5 or 6 plants per cultivar per treatment times 4 crop cycles. Letters represent mean separation comparison across lighting treatments within a cultivar using Tukey’s HSD (α = 0.05).

In terms of LA, there was a pattern for both ‘Red Oak’ and ‘Rex’ of greatest LA under the 523 nm treatment, smaller LA under the 412 nm treatment and then further declining LA as the B wavelength increased from 412 to 454 nm (especially comparing the 454 vs. 412 nm treatments) (**Figure 6A**).

For both ‘Red Oak’ and ‘Rex’ the greatest SLA (i.e., wider and thinner leaves) occurred under the 523 nm treatment. There were no significant differences observed among the B wavelength treatments with respect to SLA (**Figure 6B**).

### 3.3 Discrete blue and green light effects on chlorophyll content, colorimeter and anthocyanin

**Figure 7:**
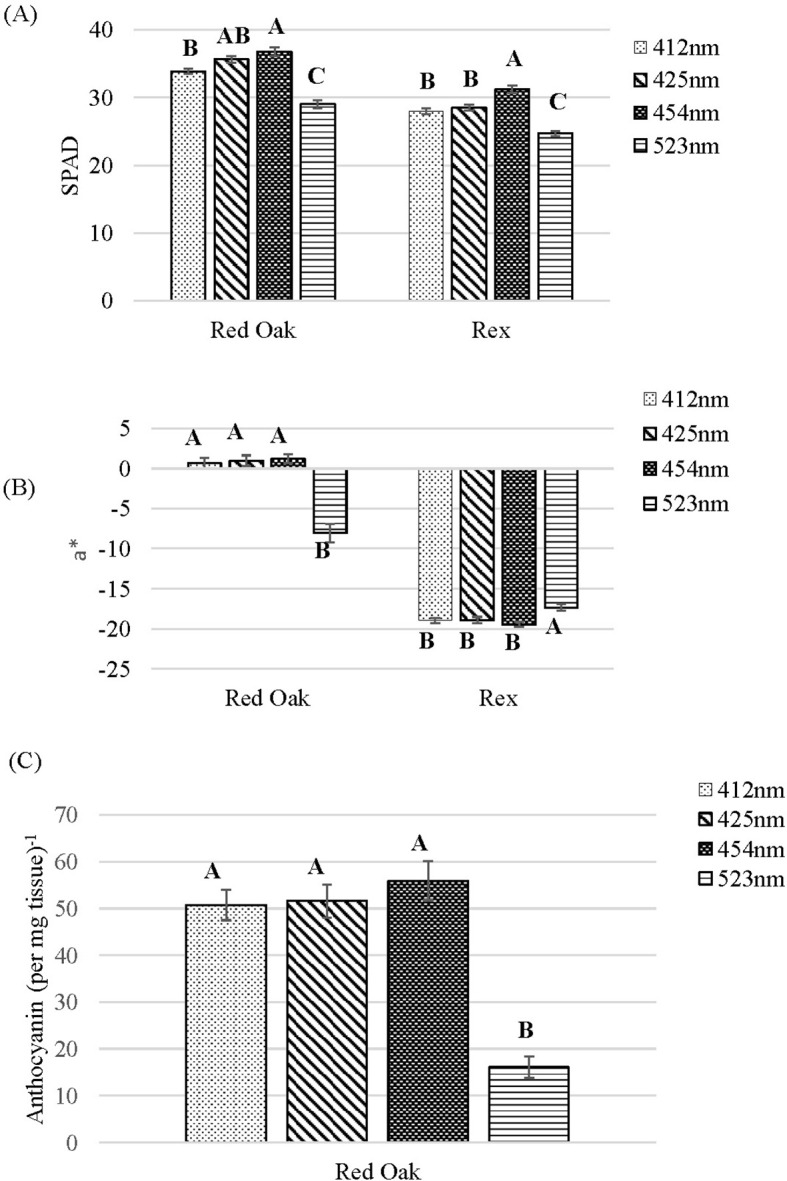
Chlorophyl content (SPAD) **(A)** and a* value **(B)** of lettuce ‘Red Oak’ and ‘Rex’ and relative anthocyanin levels per mg of tissue **(C)** of ‘Red Oak’ after 22 days of sole-source lighting treatments. Plants received 80% of their light from R (661 nm, 21 nm FWHM) and 20% of their light from either 412 nm (16 nm FWHM), 425 nm (15 nm FWHM), 454 nm (20 nm FWHM) and 523 nm (34 nm FWHM) light. Data represent means (± SE) of 5 or 6 plants per cultivar per treatment times 4 crop cycles. Letters represent mean separation comparison across lighting treatments within a cultivar using Tukey’s HSD (α= 0.05).

For chlorophyll content (SPAD index) there was a similar pattern observed for both cultivars. The lowest SPAD index was found for the 523 nm treatment which was lower than all B wavelength treatments. Within the B treatments, the SPAD index increased as the wavelength increased from 412 to 454 nm (**Figure 7A**).

The a* value measured with the colorimeter is a measure of leaf redness whereby a more positive a* value means a greater red coloration and a more negative a* value means a lower red coloration. ‘Red Oak’ under the 523 nm treatment had a significantly lower a* compared to all the B light treatments. The B light treatments (412, 425, 454 nm) resulted in statistically the same a* value (**Figure 7B**). For ‘Rex’ all B light treatments had statistically the same a* value and the values were lower than the 523 nm treatment (**Figure 7B**).

The anthocyanin content of ‘Red Oak’ leaves was statistically the same under the B light treatments. A significantly lower anthocyanin content was found for the 523 nm treatment (**Figure 7C**). The anthocyanin content of ‘Rex’ was not measured since it is not a red-leaf lettuce cultivar.

### 3.4 End-of-production blue light substitution on shoot height and width

**Figure 8:**
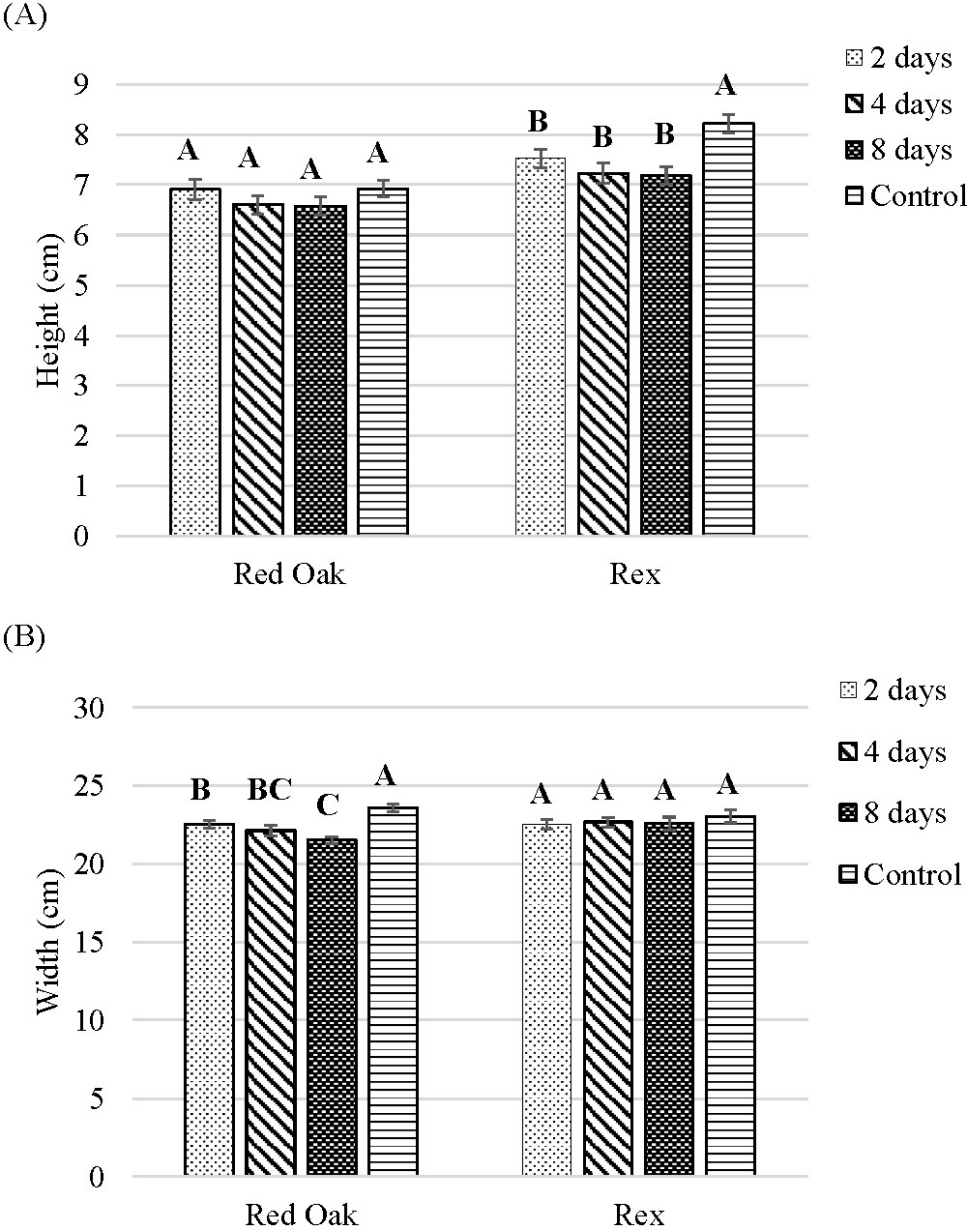
Shoot height **(A)** and width **(B)** of lettuce ‘Red Oak’ and ‘Rex’ after 22 days of sole-source lighting treatments. Control plants received 80% of their light from R (661 nm, 21 nm FWHM) and 20% of their light from G (523 nm, 34 nm FWHM) throughout a crop cycle. The 2, 4, or 8 day treatments received substitution of the G portion of light with B light (454 nm) for the last 2, 4, or 8 days of the crop cycle. Data represent means (± SE) of 5–6 plants per cultivar per treatment times 4 repeated crop cycles. Letters represent mean separation comparison across lighting treatments within a cultivar using Tukey’s HSD (α = 0.05).

**Figure 9:**
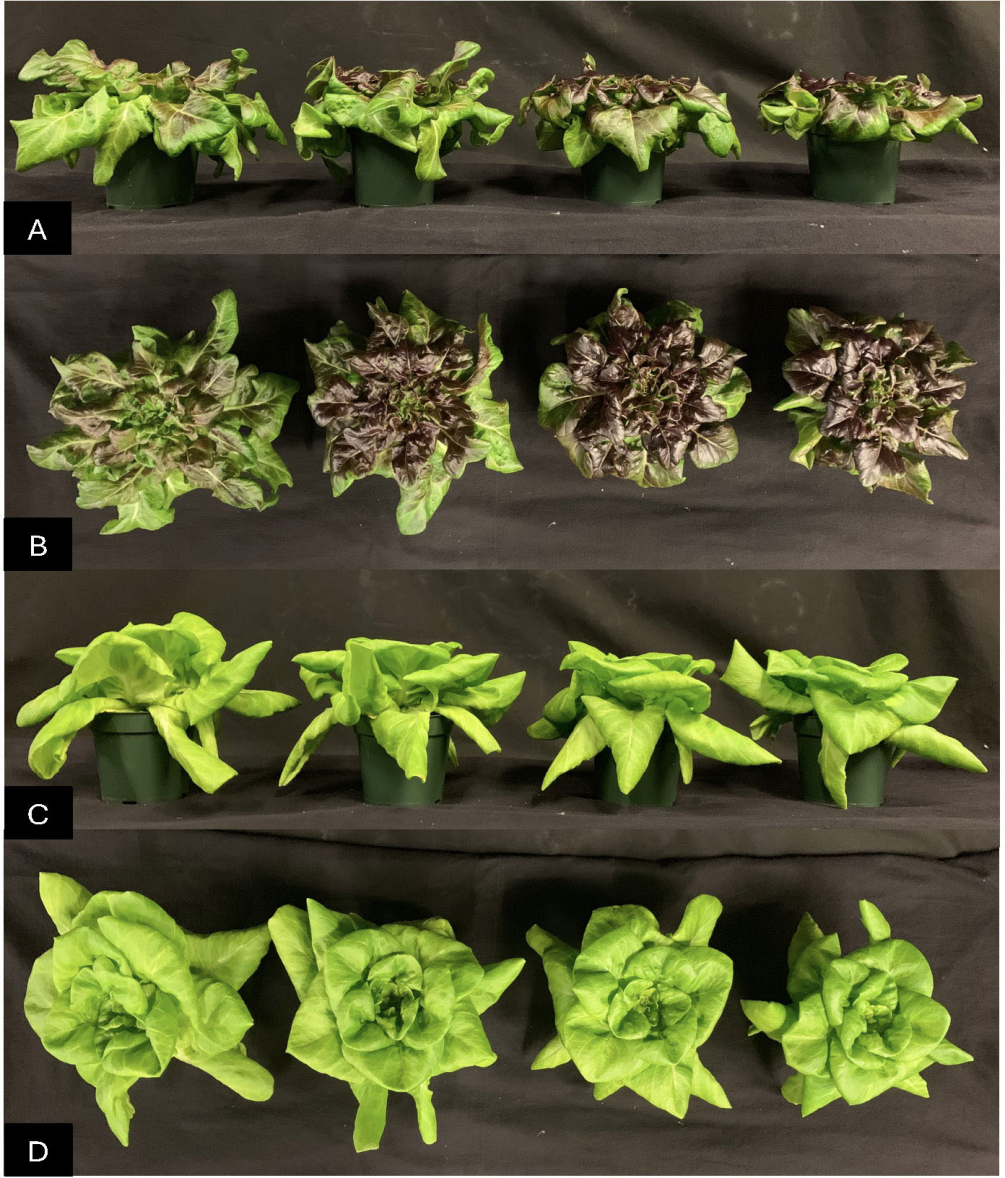
The appearance of representative ‘Red Oak’ plants from the side view **(A)**, from the top **(B)**, and ‘Rex’ plants side view **(C)** and top **(D)** after a 22-day crop cycle. From left to right plants were under 454 nm B light for 0 (control), 2, 4 or 8 days at the end of the 22-day crop cycle.

B light substitution of G light starting at 2, 4, or 8 days before the end of crop cycle did not impact the height of ‘Red Oak’. For ‘Rex’ plant height was greatest under control, and was significantly lower for the 2, 4, or 8 days of B light treatments (**Figures 8A**, **9A, C**).

Plants under the control treatment (continuous 80% R, 20% G) had the widest head for ‘Red Oak’. Two days of B light exposure before the end of the crop cycle led to significantly smaller width compared to the control treatment and increasing B exposure to 8 days further decreased the head width. For ‘Rex’, B light substitution for G light at the end of the crop cycle did not impact head width. (**Figures 8B**, **9B, D**).

### 3.5 End-of-production blue light substitution on shoot FW and DW

**Figure 10:**
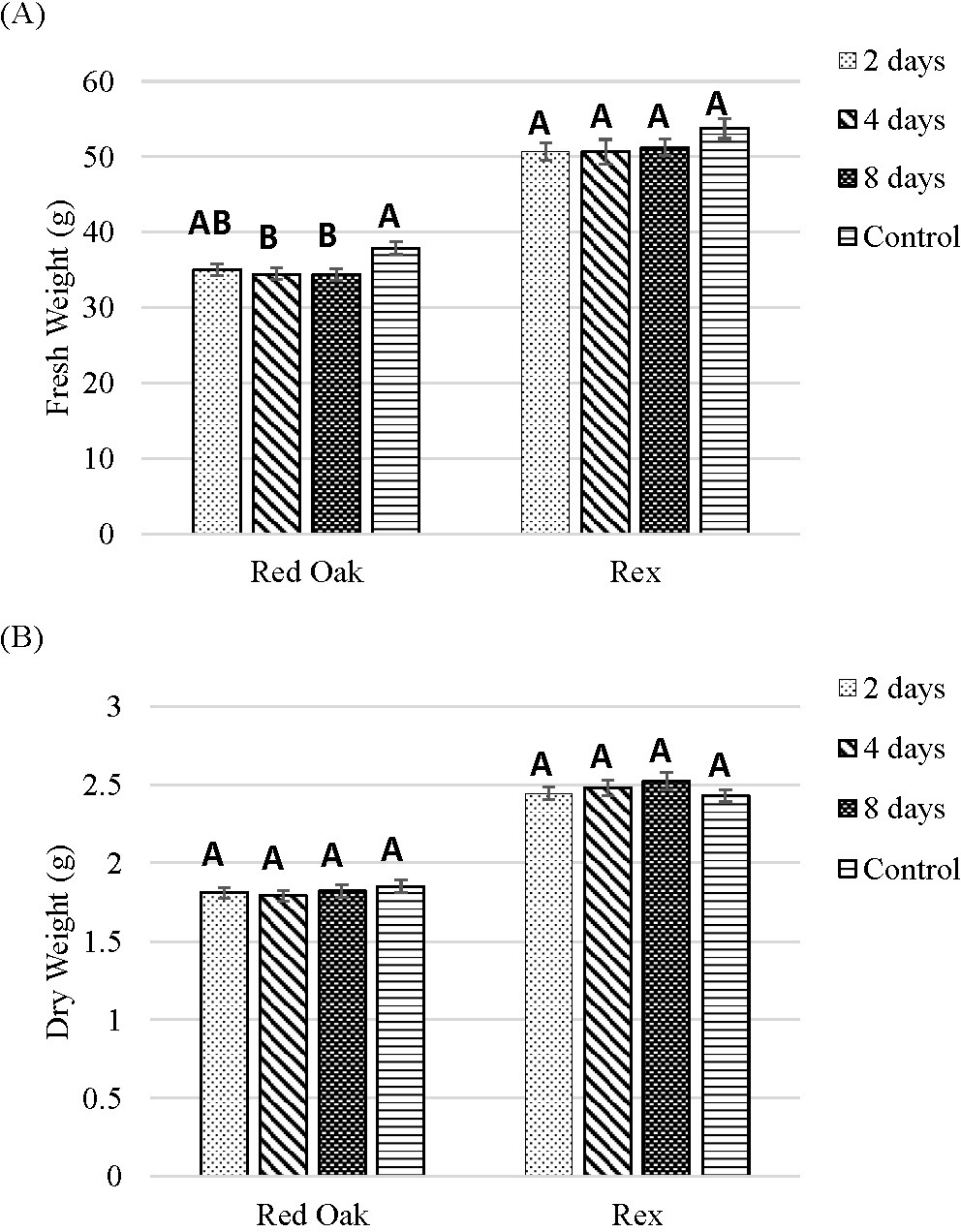
Shoot FW **(A)** and DW **(B)** of lettuce ‘Red Oak’ and ‘Rex’ after 22 days of sole-source lighting treatments. Control plants received 80% of their light from R (661 nm, 21 nm FWHM) and 20% of their light from G (523 nm, 34 nm FWHM) throughout a crop cycle. The 2, 4, or 8 day treatments received substitution of the G portion of the spectrum with B light (454 nm) for the last 2, 4, or 8 days of the crop cycle. Data represent means (± SE) of 5 or 6 plants per cultivar per treatment times 4 repeated crop cycles. Letters represent mean separation comparison across lighting treatments within a cultivar using Tukey’s HSD (α = 0.05).

For ‘Red Oak’ shoot FW was highest under the control treatment, while 4 or 8 days of B light exposure significantly decreased FW. There were no significant differences among the treatments for the FW of ‘Rex’ (**Figure 10A**).

B light substitution for G light at the end of the crop cycle did not impact the DW for either cultivar (**Figure 10B**).

### 3.6 End-of-production blue light substitution on LA and SLA

**Figure 11:**
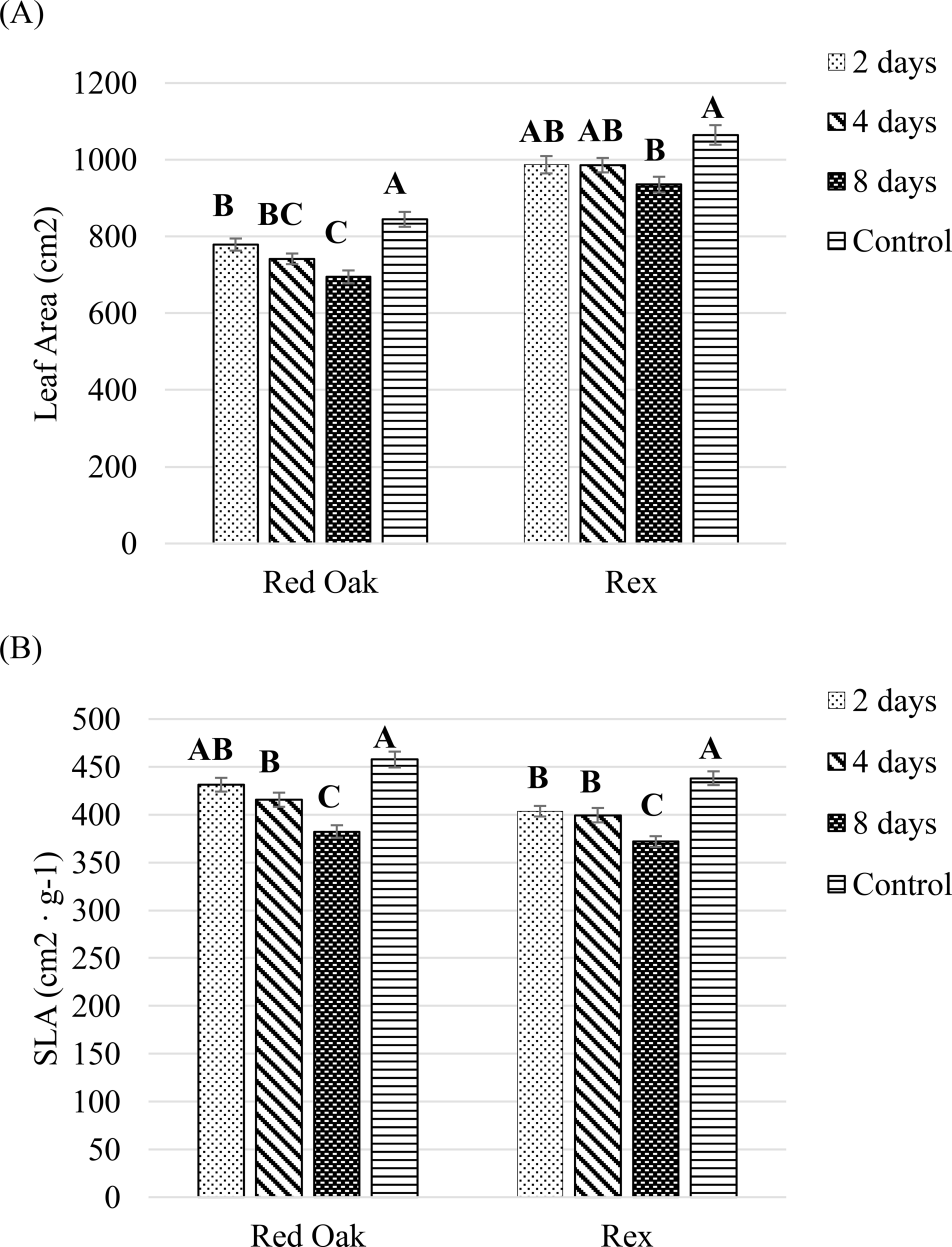
LA **(A)** and SLA **(B)** of lettuce ‘Red Oak’ and ‘Rex’ after 22 days of sole-source lighting treatments. Control plants received 80% of their light from R (661 nm, 21 nm FWHM) and 20% of their light from G (523 nm, 34 nm FWHM) throughout a crop cycle. B light (454 nm) took over a G portion of light at the different day (2, 4 and 8 days) in the end of a crop cycle as treatments. Data represent means (± SE) of 5 or 6 plants per cultivar per treatment times 4 crop cycles. Letters represent mean separation comparison across lighting treatments within a cultivar using Tukey’s HSD (α = 0.05).

For ‘Red Oak’ LA was greatest under the control treatment and decreased with increasing days of B light exposure. For ‘Rex’ only the 8 days of B light exposure resulted in significantly smaller LA compared to the other treatments (**Figure 11A**).

For SLA there was a similar pattern for both cultivars. The greatest SLA (i.e. widest/thinnest leaves) occurred under the control treatment, while the lowest SLA occurred after 8 days of B light treatment. Both the 2 and 4 days of B light treatment resulted in smaller SLA compared to the control treatment (**Figure 11B**).

### 3.7 End-of-production blue light substitution on chlorophyll content, colorimeter and anthocyanin

There was a similar pattern for both cultivars for chlorophyll content (SPAD index). The SPAD index was lowest for the control treatment and increased as the number of days of B light treatment increased (**Supplementary Figure 1A**).

For ‘Red Oak’ the a* value was lowest (i.e., least amount of R color) for the control treatment, while B light treatments for 2, 4, or 8 days at the end of the crop cycle resulted in an increased a* value (i.e., more R color) (**Supplementary Figure 1B**). For ‘Rex’ a similar result was found: 2 to 8 days of B light exposure resulted in a lower a* value compared to the control treatment.

B light exposure for 2 to 8 days increased the anthocyanin content in ‘Red Oak’ compared to the control treatment (**Supplementary Figure 1C**).

## 4 Discussion

At the end of experiment one, plants under the 20% G light (523 nm) treatment had greater biomass (FW and DW) and LA compared to the 20% 454 nm B light treatment. Those findings were similar to (Meng et al., 2019) that found substituting 449 nm B light with 526 nm G light induced shade avoidance responses of lettuce resulting in greater leaf surface area and biomass. LA is an important attribute and contributes to capturing more photosynthetically active radiation (PAR) resulting in greater carbon assimilation (Klassen et al., 2004). A novel finding in the current study was that specific wavelengths of B light radiation impact growth and development of lettuce differently. For example, while the G light treatment resulted in a greater shoot FW and DW than the 454 nm B light treatment, the biomass effects were not as evident when the plant received shorter wavelengths of B (412 or 425 nm) light. ‘Red Oak’ under the 412 nm treatment and ‘Rex’ under the 412 and 425 nm B light treatments resulted in statistically the same FW as the G light treatment although the LA was smaller. Notably, the ‘Red Oak’ exhibited biomass reductions under both 425 and 454 nm, whereas the ‘Rex’ was significantly affected only under 454 nm, indicating cultivar-specific sensitivity to discrete blue wavelengths. Chlorophyll and anthocyanin pigmentation were greater under all three blue light treatments compared with the green light treatment. Previous research confirms that anthocyanin and chlorophyll content increased as the proportion of B light increased in the total amount of radiation, but not under monochromatic B (Spalholz et al., 2020). The SPAD chlorophyll index is often used to evaluate nitrogen status and its value is highly correlated with chlorophyll content (Jiang et al., 2017; Monje and Bugbee, 1992). The chlorophyll content is correlated with the photosynthesis ratio (net photosynthesis on a LA basis) in some plants (Buttery and Buzzell, 1997; Fleischer, 1934; Kura-Hotta et al., 1987). Although chlorophyll content increased under all B treatments and was highest at 454 nm, this did not translate into greater biomass. The 454 nm treatment also produced the strongest reduction in leaf area and canopy expansion, which likely limited total canopy photon interception despite higher chlorophyll concentration per unit leaf area. Thus, whole-plant carbon gain appears to have been constrained by reduced canopy development rather than chlorophyll content alone. Chlorophyll reduction is part of the shade avoidance response due to the high % of FR (Smith and Whitelam, 1997). However, in our study the %FR was the same among treatments, but the shade avoidance response phenomena can also be triggered by G light in the absence of B light (Zhang et al., 2011). G light caused similar shade avoidance responses as would be expected from FR light treatments, however, the mechanism must be different than the FR response since %FR was kept constant in the current study. The G light response observed during the experiment is most likely caused by cryptochrome and possibly by other yet to be discovered G light receptors (Zhang et al., 2011). R and B light are both known to increase chlorophyll content through the role of the photoreceptors phytochrome A and B (R light receptors) and cryptochrome (B light receptor) (Stephenson and Terry, 2008). Thus under the G light treatment cryptochrome may become inactive due to the absence of B light leading to a lower chlorophyll content. The absorption spectra of cryptochrome might explain some of the differences observed among the B light treatments (Ahmad et al., 2002). Cryptochrome has a peak absorbance of B light at 450 nm, is less sensitive to lower wavelength B, and is not sensitive to the G light region (Bouly et al., 2007). In the current study, shorter wavelength B had less prominent B light effects (i.e., LA and biomass were not as much reduced as for the 450 nm treatment). This may reflect differential activation of cryptochrome, which has peak absorbance near 450 nm (Bouly et al., 2007), suggesting that 454 nm more strongly stimulated photomorphogenic inhibition of leaf expansion than 412 or 425 nm. In both the current study and Samuolienė et al. (2011), anthocyanin concentration responded to B light exposure. However, no significant differences were observed among the specific blue wavelengths tested.

It may be that the cryptochrome B light receptor is not sensitive to specific B wavelength in terms of anthocyanin synthesis in lettuce at least at the wavelength and intensities evaluated during this study. Furthermore, since photoperiod can interact with spectral composition in regulating developmental and circadian processes, the 18.5 h photoperiod used in the treatments may influence the magnitude of B light responses. Therefore, responses under shorter commercial photoperiods warrant further investigation.

Based on the results of experiment one, 22 days of 20% G light treatment resulted in the greatest yield (compared to 20% from 454 nm B light treatment) caused by leaf expansion, however poor leaf coloration (less chlorophyll and anthocyanin) was evident. Darker G or R leaves (based on cultivar) giving colorfulness and contrasts in salad are considered desirable by consumers (Paakki et al., 2019). In experiment 2, the substitution of G light with 454 nm B light for 2, 4, or 8 days prior to harvest improved leaf coloration in terms of red pigmentation (anthocyanin concentration) and greenness (chlorophyll content, SPAD index). The 454 nm wavelength was selected for the EoP treatment because it produced the strongest photomorphogenic responses in Experiment 1. During experiment 2 for ‘Red Oak’, substituting G light (peak at 523 nm) portion with B light (peak at 454 nm) 2 days before harvesting maximized the combination of fresh biomass and R coloration.

A study by Owen and Lopez (2015) compared 16 h of monochromatic R (659 nm; 100 μmol·m^-2^·s^-1^) or B (452 nm; 25, 50 or 100 μmol·m^-2^·s^-1^), R:B 50:50 (100 μmol·m^-2^·s^-1^), or HPS (70 μmol·m^-2^·s^-1^). This study used 4.5 μmol·m^-2^·s^-1^ LED (7:11:33:49 B:G:R:FR) as a control treatment for day length extension lighting during the end of production (starting 3, 5, 7 or 14 days before harvest) for four different cultivars of greenhouse grown lettuce. Chlorophyll content increased with supplemental lighting of 70 μmol·m^-^²·s^-^¹ HPS or 100 μmol·m^-^²·s^-^¹ of monochromatic R, B, or a 50:50 red:blue combination applied for three to fourteen days. In addition, giving at least five days of supplemental 100 μmol·m^-^²·s^-^¹ of R, B, or R:B = 50:50 lighting enhanced red pigmentation. Monochromatic supplemental B light at 25 and 50 μmol·m^-2^·s^-1^ (resulting in an additional 1.4 and 2.8 mol·m^-2^·d^-1^ of DLI) was not effective in increasing anthocyanin concentration (Owen and Lopez, 2015). Since lighting treatment supplied approximately 3.4 mol·m^-2^ B light per day in the current study, it could be assumed that 3.4 mol·m^-2^·d^-1^ B photons with a background of 80% R light are sufficient to improve R pigmentation during the last two days of the crop cycle under sole source lighting. In the current study, 8 days of end of crop cycle B light exposure negatively impacted some growth parameters. For example, in cultivar ‘Rex’ eight days of B light treatment resulted in decreased LA, but did not significantly impact width, FW and DW. For ‘Red Oak’ FW, LA, and plant width were decreased under the eight days of B light treatment (compared to the control), but B light did not impact height and DW. It should be noted that Owen and Lopez (2015) did not control for background solar radiation and differences in light intensity may confound wavelength-specific interpretations. In contrast, sole-source B light response curves reported by Cammarisano et al. (2020) provide a more controlled comparison and similarly demonstrate that increasing B light exposure can enhance pigmentation while constraining biomass accumulation.

In addition to the impact of light spectrum on plant performance, the energy efficiency of a light source must be considered in terms of industry adoption of practices. The efficacy of LED chips was measured, and these were variable based on the different wavelengths. For Philips Lumileds LEDs, G light (peak at 530 nm) had the lowest efficacy, and the efficacy of 400 nm B light was lower than that of 455 nm light (Nelson and Bugbee, 2014). Measured efficacy of our fixture showed a similar pattern, that G light had the lowest efficacy and among the B wavelengths, efficacy decreased as wavelength decreased (**Supplementary Table 1.1**). While the fixture used is for research purposes, this is also an important consideration for commercial adoption. LED technology currently has a “green gap”, i.e., there is not an optimal semi-conductor that efficiently emit G light at high current (Auf der Maur et al., 2016). Thus, while this study found 20% G light (with a background of 80% R light) can improve lettuce biomass (compared to 20% B light) it is not commercially practical to substitute B with G due to the lower efficacy. For human vision, it may be important to add G light for visual inspection. In practice, because the efficacy of dedicated G LEDs remains relatively low, G photons are often delivered indirectly through W LEDs, which are typically produced by applying a phosphor coating to B LED chips that convert part of the B emission into broader spectrum light, including G wavelengths. Adjusting specific light wavelengths can improve lettuce growth and quality; however, the economic feasibility must be evaluated before adopting these practices in a commercial setting. In this study, it was not possible to use B wavelengths longer than 454 nm. B wavelengths beyond 454 nm should be studied in future research. Further, in this study, FW of lettuce did not reach a marketable size (around 150 g) and the experiment had to be terminated at 22 days because a wider plant spacing would have stretched beyond the area lit by the lighting fixtures. Because light responses can be developmentally stage-dependent, it is possible that wavelength-specific effects observed at 22 days may change as plants approach full marketable size. While it is expected that the observed responses to light will carry through to full head size, future research should test lighting responses to B and G light until the plants reach the typical marketable size of ~150 g. While two cultivars of lettuce were tested, lighting response can be cultivar specific, and the B and G treatments should be tested on more cultivars.

## 5 Conclusion

In this study, photomorphogenic responses of lettuce were significantly influenced by light quality. Among the B light treatments B light with a peak at 454 nm resulted in the most intense stereotypical B light effects including decreased LA and internode elongation resulting in smaller plant width, FW and LA. In terms of pigmentation, chlorophyll content was the greatest for the 454 nm treatment compared to other B light treatments (with 412 and 425 nm peaks), but the anthocyanin concentration was not affected by different peak wavelengths within the B waveband. However, for both cultivars leaf coloration was poorer under G than B light treatments.

For ‘Rex’, growth parameters including FW, DW, and LA were not significantly affected by the substitution of G light with B light at the end of the crop cycle. For ‘Red Oak’, four and eight days of B light exposure at the end of the crop cycle resulted in reduced FW than the control treatment (G light for the entire crop cycle). However, two days of B light at the end of the crop cycle resulted in statistically similar FW as the control treatment. For both cultivars, leaf coloration (chlorophyll content and anthocyanin concentration) was significantly improved by B light radiation for even two days at the end of the crop cycle. Therefore, two days of B radiation at the end of the crop cycle did not decrease biomass but significantly improved crop quality in terms of the leaf coloration.

With advancements in horticultural LED technology, researchers should reconsider light quality more precisely for horticultural applications, rather than using the traditional broad wavebands for B (400 to 499 nm), G (500 to 599 nm), and R (600 to 699 nm). These results highlight the importance of precisely defining LED peak wavelength and FWHM when reporting light treatments.

## Data Availability

The raw data supporting the conclusions of this article will be made available by the authors, without undue reservation.

## Supplementary material

Supplementary Figure 1 Chlorophyl content (SPAD) **(A)**, a* value **(B)** of lettuce ‘Red Oak’ and ‘Rex’ and relative anthocyanin levels per mg of tissue **(C)** of lettuce ‘Red Oak’ after 22 days of sole-source lighting treatments. Control plants received 80% of their light from R (661 nm, 21 nm FWHM) and 20% of their light from G (523 nm, 34 nm FWHM) throughout a crop cycle. B light (454 nm) took over a G portion of light at the different day (2, 4 and 8 days) in the end of a crop cycle as treatments. Data represent means (± SE) of 5 or 6 plants per cultivar per treatment times 4 crop cycles. Letters represent mean separation comparison across lighting treatments within a cultivar using Tukey’s HSD (α = 0.05).

Supplementary Table 1 The efficacy of each channel of the LED light fixture (DYNA, Heliospectra AB, Gothenburg, Sweden). Efficacies were measured with an integrating sphere.

Supplementary Table 2 Spectral output across the 350–799 nm waveband for each treatment. Measurements were taken at six points 20 cm below the light fixture, and the averaged result is shown.
