# Supplementary figures and images for: Discrete blue and green light wavebands alter biomass, morphology, and color in lettuce (Lactuca sativa L.)
Source: Front Plant Sci. 2026 May 5;17:1735363. doi: 10.3389/fpls.2026.1735363 (PMC13184803; doi:10.3389/fpls.2026.1735363)

(A)

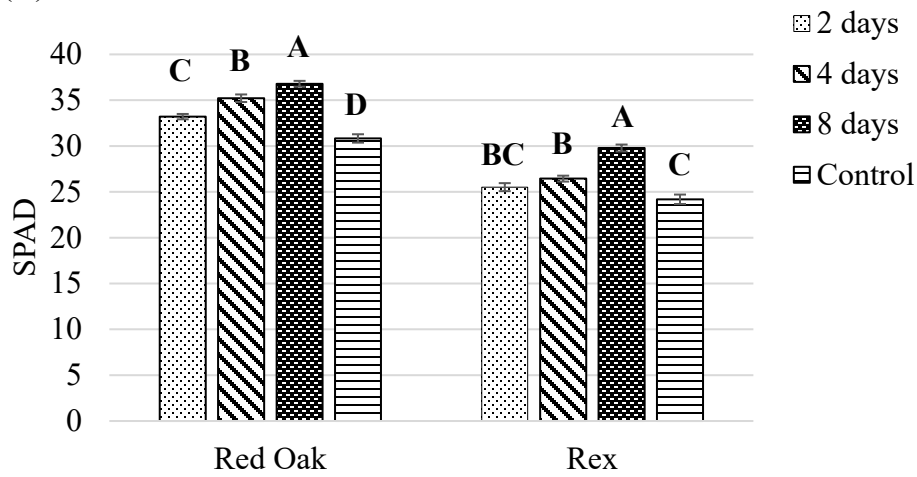

(B)

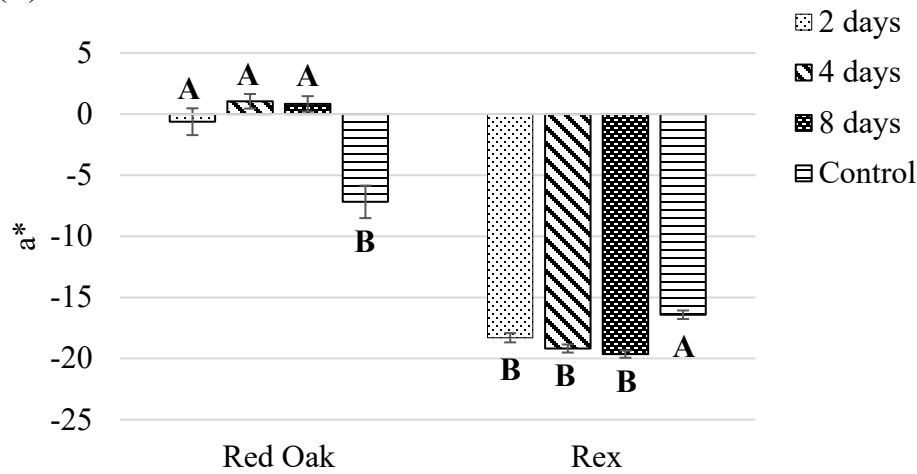

(C)

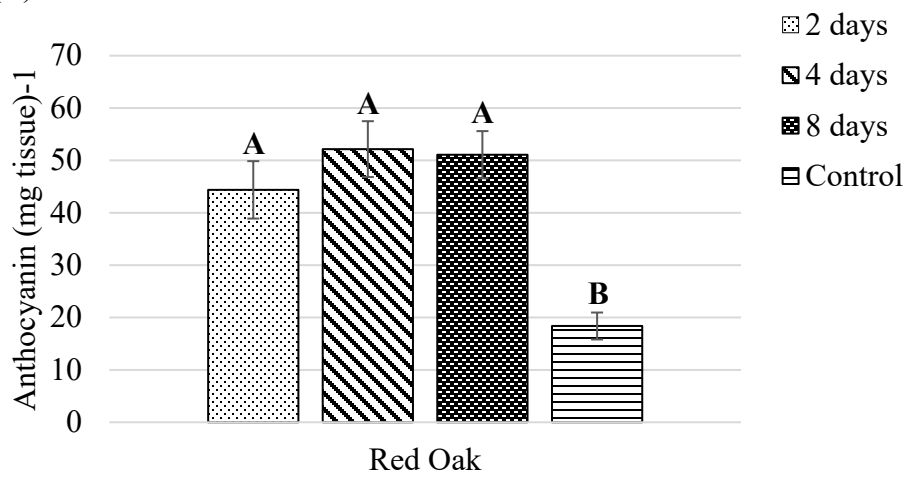

Supplement: Supplementary Figure 1 — Chlorophyl content (SPAD) (A), a* value (B) of lettuce ‘Red Oak’ and ‘Rex’ and relative anthocyanin levels per mg of tissue (C) of lettuce ‘Red Oak’ after 22 days of sole-source lighting treatments. Control plants received 80% of their light from R (661 nm, 21 nm FWHM) and 20% of their light from G (523 nm, 34 nm FWHM) throughout a crop cycle. B light (454 nm) took over a G portion of light at the different day (2, 4 and 8 days) in the end of a crop cycle as treatments. Data represent means (± SE) of 5 or 6 plants per cultivar per treatment times 4 crop cycles. Letters represent mean separation comparison across lighting treatments within a cultivar using Tukey’s HSD (α = 0.05). [file Image1.pdf]
