# Supplementary material for: Discrete blue and green light wavebands alter biomass, morphology, and color in lettuce (Lactuca sativa L.)
Source: Front Plant Sci. 2026 May 5;17:1735363. doi: 10.3389/fpls.2026.1735363 (PMC13184803; doi:10.3389/fpls.2026.1735363)
Supplement: Supplementary Table 1 — The efficacy of each channel of the LED light fixture (DYNA, Heliospectra AB, Gothenburg, Sweden). Efficacies were measured with an integrating sphere. [file Table1.docx]

| Peak wavelength (nm) | FWHM (nm) | Percent power to channel | Efficacy (µmol∙J^-1^) |
| --- | --- | --- | --- |
| 412 | 16 | 50% | 0.51 |
| 412 | 16 | 100% | 0.56 |
| 425 | 15 | 50% | 0.6 |
| 425 | 15 | 100% | 0.68 |
| 454 | 20 | 50% | 0.86 |
| 454 | 20 | 100% | 0.93 |
| 523 | 34 | 100% | 0.34 |
| 661 | 21 | 50% | 1.16 |
| 661 | 21 | 100% | 1.35 |
