# Supplementary material for: Discrete blue and green light wavebands alter biomass, morphology, and color in lettuce (Lactuca sativa L.)
Source: Front Plant Sci. 2026 May 5;17:1735363. doi: 10.3389/fpls.2026.1735363 (PMC13184803; doi:10.3389/fpls.2026.1735363)
Supplement: Supplementary Table 2 — Spectral output across the 350–799 nm waveband for each treatment. Measurements were taken at six points 20 cm below the light fixture, and the averaged result is shown. [file Table2.docx]

| Treatment | UV-A  (350-399 nm) | Blue (400-499 nm) | Green  (500-599 nm) | Red  (600-699 nm) | Far-red  (700-799 nm) | Total PAR  (µmol∙m^-2^∙s^-1^) |
| --- | --- | --- | --- | --- | --- | --- |
| 412x661 nm | 1.2% | 20.1% | 0.4% | 77.4% | 0.79% | 247 |
| 425x661 nm | 0.2% | 21.0% | 0.4% | 77.4% | 0.89% | 256 |
| 454x661 nm | 0.1% | 20.3% | 0.5% | 78.4% | 0.78% | 251 |
| 523x661 nm | 0.1% | 1.3% | 18.7% | 79.1% | 0.80% | 262 |
